# Health-Related Quality of Life and Emotional Health in X-Linked Carriers of Chronic Granulomatous Disease in the United Kingdom

**DOI:** 10.1007/s10875-019-00607-6

**Published:** 2019-03-13

**Authors:** Alexandra C. Battersby, Helen Braggins, Mark S. Pearce, Fiona McKendrick, Mari Campbell, Siobhan Burns, Catherine M. Cale, David Goldblatt, Andrew R. Gennery

**Affiliations:** 1 Great North Children’s Hospital, Clinical Resource Building, Floor 4, Block 2, Queen Victoria Road, Newcastle upon Tyne, NE1 4LP UK; 2 Department of Immunology, Great Ormond Street Hospital, London, UK; 3 Institute of Health and Society, Newcastle University, Newcastle upon Tyne, UK; 4 Department of Immunology, Royal Free Hospital, London, UK; 5 UCL Institute of Immunity and Transplantation, London, UK; 6 Institute of Child Health, University College London, London, UK; 7 Institute of Cellular Medicine, Newcastle University, Newcastle upon Tyne, UK

**Keywords:** X-linked chronic granulomatous disease carrier, health-related quality of life, anxiety, depression

## Abstract

X-linked chronic granulomatous disease (XL-CGD), a rare primary immunodeficiency due to a defect in the gp91^phox^ NADPH oxidase subunit, results in recurrent, severe infection, inflammation, and autoimmunity. Patients have an absent, or significantly reduced, neutrophil oxidative burst. Due to lyonization, XL-CGD carriers have a dual population of functional and non-functional phagocytes and experience a range of symptoms including increased risk of autoimmunity, fatigue, and infection. Patients with CGD have poorer quality of life (QoL) than normal controls. We evaluated QoL and psychological health in UK XL-CGD carriers. Recruited participants completed the Medical Outcomes Study Short Form 36 version 2 (SF-36 V2), providing an overall score for mental and physical health. Psychological health was assessed using the Hospital Anxiety and Depression Scale (HADS) questionnaire. Seventy-five XL-CGD carriers were recruited from 62 families, median age 43 years (range 3–77). Fifty-six were mothers, 6 grandmothers, and 13 siblings. Sixty-two completed the SF36v2 and had reduced QoL scores compared with adult CGD patients and a UK age-matched female control cohort, indicating a reduced QoL. Sixty-one completed a HADS questionnaire. Over 40% experienced moderate or greater levels of anxiety with only one third being classified as normal. Higher anxiety scores significantly correlated with higher depression scores, lower self-esteem, presence of joint or bowel symptoms, and higher levels of fatigue (*p* < 0.05). This is the first study to evaluate QoL of XL-CGD carriers, and demonstrates high rates of anxiety and significantly reduced QoL scores. XL-CGD carriers should be considered as potential patients and pro-actively assessed and managed.

## Introduction

Chronic granulomatous disease (CGD) is a rare inborn error of immunity in which a defect in one of the subunits of NADPH oxidase results in a defective respiratory burst of phagocytic cells, causing recurrent infection, inflammation, and autoimmunity [1]. Patients with CGD who continue on maintenance prophylactic treatment have poorer quality of life (QoL) than healthy controls or CGD patients who underwent curative hematopoietic stem cell transplantation [2].

CGD can be X-linked or autosomal recessive. Worldwide, X-linked (XL) cases due to defects in the gp91^phox^ subunit are most common, and in the United Kingdom (UK), 70% of cases are X-linked (XL) [3]. We, and others, have previously described a range of symptoms in XL-CGD carriers. This includes an increased risk of autoimmunity (in particular systemic lupus erythematosus (SLE)–like symptoms), fatigue, and, in some cases, infection, attributed to their dual population of functional and non-functional phagocytes [4–6]. Thus, there is a range of factors that may affect the QoL of XL-CGD carriers. In addition to the symptoms above, they also have risk factors around caring for a family member with chronic disease and symptoms of anxiety and depression that may be associated with SLE. We aimed to evaluate QoL and psychological health in XL-CGD carriers in the UK.

## Methods

XL-CGD carriers were identified through the UK CGD Registry and approached either in person or through the mail. Girls under the age of 16 years who did not know their carrier status were not included in the study.

Carriers who were aware of their carrier status were eligible and included where appropriate, irrespective of their age. In families where the index case was deceased, families were contacted if the physician caring for the family deemed it appropriate or if there was continuing contact with the family.

To assess health-related QoL, recruited participants completed the Medical Outcomes Study Short Form 36 version 2 (SF-36 V2), which is a validated QoL questionnaire [7]. The SF36v2 provides a numerical score for 8 domains, along with an overall score for mental health and physical health. Lower scores indicate poorer QoL. If the index case had undergone HSCT, the SF-36 for the XL-CGD carrier was delayed until a year had passed since the procedure. HSCT is an intense period for family and patient alike, and the associated anxiety and distress, whilst transient, may be evident for up to 6 months after the transplant is completed [8, 9].

To assess psychological health, participants completed questionnaires about self-esteem, and presence of anxiety and depression symptoms, using the Hospital Anxiety and Depression Scale (HADS) [10], a questionnaire comprising 14 questions: 7 assessing anxiety and 7 assessing depression. The score generated after completion of the questionnaire, for both anxiety and depression was categorized and compared with population data [11] and scores from patients with SLE [12, 13] and carers of patients with cystic fibrosis [14].

Statistical analysis was carried out using STATA® (StataCorp LLC, TX, USA). Data from the QoL questionnaires were inputted into the licenced software, which generated total scores for each domain. Each domain could score a maximum of 100. Scores were compared by the software with population data to produce norm-based scores (NBS). Spearman’s correlation was used to assess factors associated with anxiety and depression scores.

## Results

Eighty families were identified from the UK CGD register, and 75 XL-CGD carriers were recruited from 62 families. The median age of participants was 43 (range 3–77) years. Fifty-six were mothers, 6 grandmothers, and 13 siblings. In 8 families, the index case had died, but only 5 questionnaires were returned from these families, too few to facilitate a separate analysis.

**Fig. 1:**
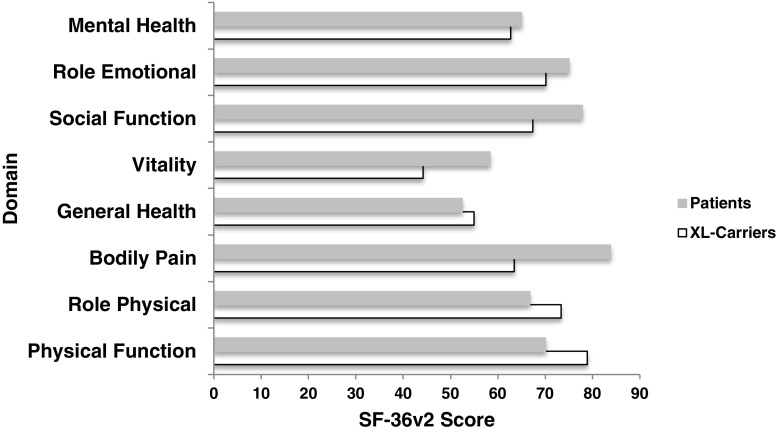
Quality of life scores in X-linked chronic granulomatous disease carriers compared with adult chronic granulomatous disease patients (100 = normal)

Sixty-two participants completed the SF36v2, of which 55 (70%) were mothers of an index case. XL-CGD carriers had similar, reduced QoL scores to adult CGD patients (Theresa Cole, personal communication) and scored lower than CGD patients in 4 domains (vitality, emotional, social function, and mental health) (Fig. 1). Compared with a UK age-matched female control cohort, the scores for XL-CGD carriers were significantly worse, indicating a reduced QoL (Table 1). There was no significant difference in scores between carrier mothers and other carrier relatives in any domain (Table 2). There were significant associations between the SF36 Physical Component summary score and all physical symptoms except the skin manifestations of photosensitivity and recurrent abscesses. There was a significant association with degree of oxidative function, but no age-related association. There were significant associations between the SF36 Mental Component summary score and joint pains, gastrointestinal and respiratory symptoms, number of fulfilled ARA criteria, and recurrent skin abscesses. We found no correlation with age of participant or degree of oxidative function. There was no relationship between physical or mental quality of life and transplant status of the index case.

**Table 1:** Mean SF36v2 scores in XL-CGD carriers compared with UK population data (100 = normal)

Domain	CGD carriers	UK norms (female age 35–54) [15]	*p* value
Physical function	79.28 (29.4)	89.4 (18.3)	< 0.001
Role physical	74.35 (32.2)	84.0 (32.0)	0.00235
Bodily pain	66.24 (30.5)	79.4 (22.0)	0.002
General health	56.31 (28.5)	74.1 (20.3)	< 0.001
Vitality	44.98 (25.5)	58.2 (19.9)	0.002
Social function	69.68 (29.5)	86.7 (20.5)	0.001
Role emotional	71.88 (31.3)	80.3 (33.6)	0.0341
Mental health	63.16 (16.8)	71.6 (17.8)	0.005

**Table 2:** Correlation coefficient and *p* value for Mental Component and Physical Component scores of quality of life with symptoms, degree of neutrophil function, and age

	MCS	PCS
Joint symptoms	− 0.33 **0.0085**	− 0.42 **0.0009**
IBD score (bowel symptoms)	− 0.69 **< 0.0001**	− 0.50 **< 0.0001**
Respiratory score	− 0.32 **0.016**	− 0.56 **< 0.0001**
Photosensitivity	− 0.12 0.36	− 0.18 0.17
Recurrent abscesses	− 0.34 **0.0074**	− 0.14 0.27
Ulcers	−0.14 0.30	− 0.39 **0.002**
Number of ARA criteria	− 0.31 **0.016**	− 0.39 **0.002**
% NOB	0.18 0.29	0.44 **0.005**
Age of participant	− 0.55 0.67	− 0.23 0.07
Relationship to index case	−0.049 0.71	− 0.046 0.73
Index case undergone HSCT	− 0.13 0.45	0.17 0.32

*MCS* SF36 Mental Component summary score, *PCS* SF36 Physical Component summary score, *IBD* inflammatory bowel disease, *ARA* American Rheumatism Association, *NOB* neutrophil oxidative burst, *HSCT* hematopoietic stem cell transplantation

Bold entries are significant *p* values

Sixty-one participants returned a completed HADS questionnaire. The frequency of a pre-existing diagnosis of anxiety or anxiety and depression is shown (Table 3). Only 1 XL-CGD carrier suffered from isolated anxiety, but a greater number had a diagnosis of mixed anxiety and depression. Twelve XL-CGD carriers had been prescribed antidepressants. Over 40% of XL-CGD carriers suffered from moderate or greater levels of anxiety with only one third being classified as normal (Table 4). The distribution of anxiety categories was similar, irrespective of the relationship of the carrier to the index case. Higher anxiety scores were significantly correlated with higher depression scores, lower self-esteem, the presence of joint or bowel symptoms, and higher levels of fatigue (*p* < 0.05). There was no significant correlation of high anxiety scores with age, relationship to the index case, or a diagnosis of SLE in the carrier (*p* > 0.05). There was no significant difference in mean anxiety scores of the XL-CGD carriers when considered with regard to age categories of the index case or when compared with whether the index case had undergone HSCT or remained on conventional therapy. There was no significant difference in anxiety scores when compared with published data from SLE patients [13].

**Table 3:** Pre-existing anxiety and depression diagnoses and treatment

Diagnosis	Number of XL-CGD carriers	% affected
Anxiety	1	1.6
Depression	7	11.6
Mixed anxiety and depression	6	10
Prescribed antidepressants	12	20

**Table 4:** Anxiety and depression categories in XL-CGD carriers

HAD anxiety category (score)	Number of carriers (%)	HAD depression category (score)	Number of carriers (%)
Normal (0–7)	21 (34.4)	Normal (0–7)	45 (73.8)
Mild (8–10)	14 (23)	Mild (8–10)	11 (18)
Moderate (11–14)	22 (36.1)	Moderate (11–14)	4 (6.6)
Severe (> 14)	4 (6.5)	Severe (> 14)	1 (1.6)
Total	61 (100)	Total	61 (100)

With respect to the depression component, nearly three quarters of the cohort were categorized as normal, with only 1 XL-CGD carrier in the severe category (Table 4). Depression was considerably less prevalent than anxiety in the XL-CGD carriers. Higher depression scores were significantly correlated with higher anxiety scores, lower self-esteem, and higher fatigue scores. There was no significant correlation of depression scores with age, relationship to index case, and clinical symptoms (gastrointestinal and joint symptoms, diagnosis of SLE-like disorder, and number of ARA SLE criteria met).

A comparison of HAD scores was made with published data from carers of children with cystic fibrosis [14], a disease which is inherited in an autosomal recessive manner (Table 5). This demonstrated that anxiety scores were significantly higher in XL-CGD carriers than the cystic fibrosis parents, whilst the depression scores were higher but did not reach significance.

**Table 5:** Comparison and anxiety and depression scores in XL-CGD carriers with published data of carers of cystic fibrosis

	XL-CGD carriers	SLE (high pain) [12]	SLE (low pain) [12]	SLE [13]	CF carers [14]
Number	60	20	64	120	650
Age (years)	42.5	45.9	45.9	38	40.35
Depression score (mean)	5.08	8	3	6	4.36
*p* value		1.0	< 0.01	0.98	0.084
Mean anxiety score	9.54	9	4	9	7.52
*p* value		0.18	< 0.01	0.18	0.0002

## Discussion

For patients affected by primary immunodeficiencies (PID), research has primarily concentrated on elucidating the pathophysiology of the disease, conducting epidemiological studies to define the prevalence in different populations, and determining the prognosis with different treatment modalities that are available. However, for patients, an important aspect of the disease is the impact it has upon daily living. An association between a reduced quality of life and chronic disease is well recognized [16–18]. Previously, we have demonstrated improvements in quality of life in patients with CGD who had successfully undergone curative treatment with hematopietic stem cell transplantation, compared with those who continued with conservative prophylactic antimicrobial and anti-inflammatory treatment [2]. Furthermore, we, and others, have demonstrated that many carriers of X-linked CGD experience significant inflammatory, autoimmune, and more rarely infectious symptoms as a result of lyonization, leading to dual neutrophil populations exhibiting normal and diminished or absent function associated with inflammation [4, 5]. Our study is the first to investigate health-related quality of life and emotional health in any cohort of carriers of a primary immunodeficiency. Given that we now appreciate that X-linked CGD carriers have their own mutated gene–related disease issues, it is perhaps not surprising that many of them exhibit a reduced health-related quality of life, similar to that of adult CGD patients, and worse than UK normal controls.

There are many factors that may impact upon the psychological health of XL-CGD carriers, including being a caregiver for a child with chronic illness, genetic guilt, the presence of anxiety and depressive symptoms, and potential ill health of the subject themselves. Whilst there is no literature specifically about XL-CGD carriers in this area, there is literature from other conditions, which may be relevant to XL-CGD carriers. Caring for a child with a chronic illness increases levels of stress. It is less clear what that impact may be on other markers of psychological health including the presence of anxiety and depression. Having a child with a chronic condition impacts upon the psychological health of the family [19]. Parental stress has been evaluated following HSCT of a child for malignant disease and primary immunodeficiency [20]. Mothers were more prone to general stresses even 5 years after their child’s HSCT but do not report higher stress scores when compared with reference groups. Genetic guilt may account for some of the psychological distress seen in the carers of genetic disorders, but if this were the main cause, one would expect to see lower levels of distress in conditions where there is no such definitive inheritance. This does not appear to be true, as a study of pediatric inflammatory bowel disease patients and their parents found high levels of caregiver stress, with highest levels seen where the disease was most active [21]. We have previously demonstrated that fatigue in carriers does not correlate with the disease status of the index case, as those with sons who had been successfully transplanted also exhibited symptoms [6], suggesting that intrinsic factors also play a part. In this study, symptoms were found in siblings, which were no different to those in mothers or grandmothers, again suggesting that intrinsic factors play a role, rather than “genetic guilt.” Our findings of significantly reduced QoL in XL-CGD carriers compared with UK population norms, and comparable with that of adult CGD patients, are important, as it highlights an unrecognized and unmet need that needs to be addressed in these women. Whilst there are many factors that may have contributed to these findings, an increased awareness of these findings should aid clinicians in caring for the entire family, including female relative carriers, and to institute appropriate measures as indicated [22]. It would be interesting to study QoL in other X-linked primary immunodeficiencies. In that respect, chronic granulomatous disease may be unique—to date, it seems to be the only disease where XL carriers may exhibit symptoms regardless of degree of lyonization. Furthermore, CGD is one of few diseases where there may be a reasonable mix between transplanted and non-transplanted patients—in Wiskott-Aldrich syndrome, common gamma chain–deficient severe combined immunodeficiency, and X-linked lymphoproliferative disease, the majority of patients are transplanted or deceased. Perhaps CD40L-deficiency is most similar in this regard, although carriers appear asymptomatic—it is however less common than XL-CGD, and gathering meaningful cohorts of patients and carriers will be more challenging.
